# Sum-Rate of Multi-User MIMO Systems with Multi-Cell Pilot Contamination in Correlated Rayleigh Fading Channel

**DOI:** 10.3390/e21060573

**Published:** 2019-06-06

**Authors:** Menghan Wang, Dongming Wang

**Affiliations:** 1 National Mobile Communications Research Laboratory, Southeast University, Nanjing 210096, China; 2 Purple Mountain Laboratories, Nanjing 211111, China

**Keywords:** multi-user MIMO, pilot contamination, imperfect channel estimation, correlated Rayleigh fading, sum-rate analysis

## Abstract

This paper presents some exact results on the sum-rate of multi-user multiple-input multiple-output (MU-MIMO) systems subject to multi-cell pilot contamination under correlated Rayleigh fading. With multi-cell multi-user channel estimator, we give the lower bound of the sum-rate. We derive the moment generating function (MGF) of the sum-rate and then obtain the closed-form approximations of the mean and variance of the sum-rate. Then, with Gaussian approximation, we study the outage performance of the sum-rate. Furthermore, considering the number of antennas at base station becomes infinite, we investigate the asymptotic performance of the sum-rate. Theoretical results show that compared to MU-MIMO system with perfect channel estimation and no pilot contamination, the variance of the sum-rate of the considered system decreases very quickly as the number of antennas increases.

## 1. Introduction

Massive multiple-input multiple-output (MIMO) antenna technology has emerged as an effective technique for significantly improving the capacity of wireless cellular systems, since the pioneering works in [1,2,3]. When base station (BS) is equipped with large-scale antenna array, large number of users can be served in the same time-frequency resource. From the theoretical point of view, when BS has perfect channel state information (CSI) of its own users, the sum-rate of multi-user MIMO (MU-MIMO) system can be arbitrary large as the number of antennas at BS becomes infinite. However, a bottleneck of massive MIMO is the acquisition of CSI. Usually, the pilot number is linear increasing with the number of users, even for time-division duplex system. Due to the limitation of the pilot resource, multi-cell multi-user orthogonal pilot design is not practical in massive MIMO system. Thus, pilot contamination due to pilot reuse in adjacent cells becomes a new character of massive MIMO [4,5,6]. Some recent work [7,8] proposes efficient schemes to reduce the effect of inter-cell interference caused by pilot contamination in massive MIMO systems. In this paper, we focus on the analysis of the capacity performance for MIMO systems subject to pilot contamination.

### 1.1. Differences and Motivation (Regarding the Related Work)

The capacity performance for MU-MIMO with the pilot contamination has been studied under the independently and identically distributed (i.i.d.) Rayleigh fading channels [9]. However, for practical systems, when the number of antennas is very large, the channels are not i.i.d. usually. In [10,11,12], the spectral efficiency of MU-MIMO with multi-cell pilot contamination was analyzed under correlated Rayleigh fading channels with minimum-mean-squared-error (MMSE) channel estimator. In [10], only the target cell channel parameters were estimated at the BS, and the expectation of the covariance matrix of extrinsic cell interference was used to simplify the performance analysis for linear receiver/precoder. As the Remark 2.2 of [11] mentioned, estimation of all of extrinsic cell interference channel matrices could further improve the performance. In [13], this problem has been considered for i.i.d. Rayleigh fading channels. The optimal linear receiver considered the correlation between the channel estimates and the inter-cell interference was proposed to improve system performance. However, H [13] only derived an approximate expression of the sum-rate. Furthermore, to the best of the authors’ knowledge, most of the current research only focuses on the ergodic capacity of multi-cell MU-MIMO with pilot contamination. The performance of the outage capacity has not been investigated.

### 1.2. Our Contribution

In this paper, we focus on the sum-rate of MU-MIMO with multi-cell pilot contamination under correlated Rayleigh fading channels. The closed-form expressions for the statistical of the sum-rate are given. The contributions of this work are summarized as:

- For a finite number of BS antennas, we derive closed-form expression of the moment generating function (MGF) for the lower bound of the sum-rate. Then, we obtain the ergodic sum-rate and the approximated variance of the sum-rate. With Gaussian approximation, outage sum-rate and outage probability are also studied in this paper.
- We investigate the asymptotic performance of the sum-rate. When the number of antennas at BS approaches infinite, the variance of the sum-rate of MU-MIMO systems with perfect channel estimation and no pilot contamination decreases as 1/M [14], where *M* is the number of antennas at BS. In this paper, we show that the variance of the sum-rate of MU-MIMO systems with imperfect CSI and multi-cell pilot contamination decreases as $1/M^{3}$.

### 1.3. Notation

The notations adopted in this paper conform to the following convention. Column vectors are denoted in lower case bold: x. Matrices are upper case bold: A. $I_{M}$ denotes the identity matrix of M×M. 0 denotes an all-zero matrix (or vector), and its size depends on the context. ${\left[A\right]}_{i,j}$ denotes the (i,j)th entry of a matrix A. ${\left(\cdot \right)}^{H}$ represents the Hermitian transpose. Tr(A) and det(A) denote the trace and the determinant of A, respectively. $∥\cdot ∥$ denotes the spectral norm of a matrix. The operator ln(·) denotes the natural logarithm. The operators E(·) and V(·) denote expectation and variance, respectively. The covariance operator is given by $\mathrm{cov}\left(x,y\right)≜E\left(xy^{H}\right)-E\left(x\right)E\left(y^{H}\right)$. The distribution of a complex Gaussian random variable with zero mean and variance $\sigma^{2}$ is denoted as $N\left(0,\sigma^{2}\right)$. CN(0,Σ) stands for zero mean circularly-symmetric complex Gaussian (ZMCSCG) distribution with covariance matrix Σ. $\overset{a.s.}{\to }$ is the almost sure (a.s.) convergence and $\overset{d}{\underset{\;\;\;}{\to }}$ means convergence in distribution. erfc(·) is the complementary error function defined by

$$\mathrm{erfc}\left(x\right)=\frac{2}{\pi }\int_{x}^{\infty }e^{-t^{2}}dt,$$

and ${\mathrm{erfc}}^{-1}\left(\cdot \right)$ is the inverse complementary error function.

## 2. Equivalent System Model of Multi-Cell MU-MIMO with Pilot Contamination

**Figure 1:**
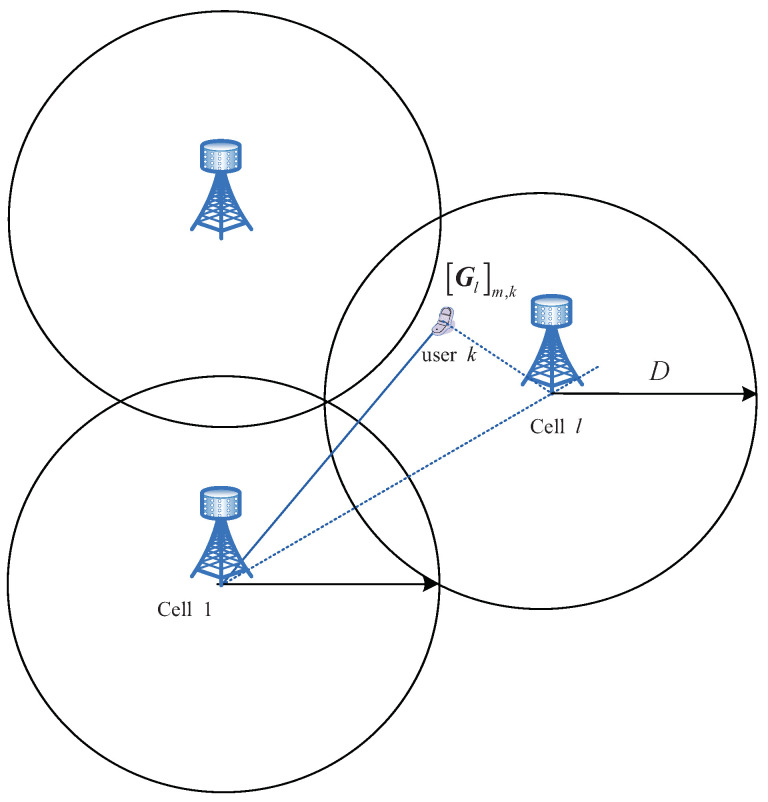
Multi-cell multi-user multiple-input multiple-output (MU-MIMO) system (there are three BSs, each of which is equipped with *M* antennas, and the mobile is equipped with one antenna).

Figure 1 shows a multi-cell MU-MIMO system. The frequency reuse factor is set to be one in this paper. Consider a cellular system with *L* time-synchronized cells and the *L* cells share the same frequency band. Here, we assume that all base stations are time-synchronized using high-precision global positioning system (GPS) or IEEE 1588 precision time protocol [15]. Each cell contains one base station equipped with *M* antennas and *K* single-antenna users. Throughout the paper, we assume M≥K. To simplify the notations, we assume cell 1 is the reference cell (the BS of cell 1 is the reference BS).

We consider uplink transmission. At the reference BS, the M×1 received vector is given by

(1) $$y_{1}=G_{1}x_{1}+\sum_{l=2}^{L}G_{l}x_{l}+z_{1},$$

where $x_{l}∼\mathrm{CN}\left(0,\gamma_{U}I_{K}\right)$ is the overall transmitted signal vector of cell *l*, $z_{1}∼\mathrm{CN}\left(0,\gamma_{U}I_{M}\right)$, $1/\gamma_{U}$ denotes the uplink signal-to-noise ratio (SNR), and $G_{l}$ represents the M×K channel matrix between the *K* users in cell *l* and the reference BS, i.e., ${\left[G_{l}\right]}_{m,k}$ is the channel coefficient between the *k*th user in cell *l* and the *m*th antenna of the reference BS. We model $G_{l}$ as

(2) $$G_{l}=\lambda_{l}^{\frac{1}{2}}R^{\frac{1}{2}}H_{l},$$

where $\lambda_{l}\overset{\Delta }{\;=}cd_{l}^{-\alpha_{l}}s_{l}$ denotes the large-scale fading between all of the users in cell *l* and the reference BS, and $\alpha_{l}$ is the path loss exponent, typically between 3.0 and 5.0. Moreover, *c* is the median of the mean path gain at a reference distance $d_{l}=1\;\mathrm{km}$, and $s_{l}$ is a log-normal shadow fading variable. $H_{l}$ denotes the small scale fading, and each entry of $H_{l}$ is an i.i.d. ZMCSCG random variable of unit variance. R is the deterministic receive correlation matrix at the reference BS, which satisfies the following hypothesis [10]:

**Hypothesis** **1.**

*1)*  *$\underset{M\to \infty }{\lim\sup}∥R∥<\infty$, where limsup denotes the limit superior.*
*2)*  *$\underset{M\to \infty }{\lim\inf}\frac{1}{M}\mathrm{Tr}\left(R\right)>0$, where liminf denotes the limit inferior.*

Similar to T [9], the same pilot sequence set is reused among all of the cells. To achieve better performance, in each cell, we adopt an orthogonal pilot sequence set. Without loss of generality, during the coherent time interval, it is also assumed that the minimum number of pilot symbols is adopted. With these assumptions, the received pilot signal at the reference BS can be given by

(3) $$\begin{array}{c} Y_{P}=\sum_{l=1}^{L}G_{l}+Z_{P}, \end{array}$$

where $Y_{P}$ is an M×K received pilot signal matrix, $Z_{P}$ is an M×K noise matrix and each element is i.i.d ZMCSCG random variable with variance $\gamma_{P}$, and $1/\gamma_{P}$ is the training SNR.

Given the linear model in Equation (3), the channel matrix $G_{l}$ can be decomposed as

(4) $$\begin{array}{c} G_{l}={\hat{G}}_{l}+{\tilde{G}}_{l}, \end{array}$$

where ${\hat{G}}_{l}$ denotes the estimation of $G_{l}$, and ${\tilde{G}}_{l}$ denotes the channel estimation error. With MMSE estimator [16], ${\hat{G}}_{l}$ can be computed as

(5) $$\begin{array}{c} {\hat{G}}_{l}=\lambda_{l}RQ^{-1}Y_{P}, \end{array}$$

where Q is defined by

(6) $$\begin{array}{c} Q≜\left(\sum_{l=1}^{L}\lambda_{l}\right)R+\gamma_{P}I_{M}. \end{array}$$

Defining

$$\hat{H}≜Q^{-\frac{1}{2}}Y_{P},$$

the entries of $\hat{H}$ are also i.i.d. ZMCSCG with unit variance. Thus, ${\hat{G}}_{l}$ can be modeled by

(7) $$\begin{array}{c} {\hat{G}}_{l}=\lambda_{l}RQ^{-\frac{1}{2}}\hat{H}. \end{array}$$

By the property of MMSE estimation, ${\hat{G}}_{l}$ and ${\tilde{G}}_{l}$ are uncorrelated, and the entries of ${\tilde{G}}_{l}$ are ZMCSCG with

(8) $$\begin{array}{c} E\left({\tilde{G}}_{l}{\tilde{G}}_{l}^{H}\right)=K\left(\lambda_{l}R-\lambda_{l}^{2}RQ^{-1}R\right). \end{array}$$

Substituting $G_{l}$ in Equation (1) with Equation (4) yields

(9) $$\begin{array}{c} y_{1}={\hat{G}}_{1}x_{1}+\sum_{l=2}^{L}{\hat{G}}_{l}x_{l}+{\tilde{z}}_{1}, \end{array}$$

where

$${\tilde{z}}_{1}≜\sum_{l=1}^{L}{\tilde{G}}_{l}x_{l}+z_{1}.$$

The covariance matrix of ${\tilde{z}}_{1}$, denoted as Σ, can be computed as

(10) $$\begin{array}{c} \Sigma =\mathrm{cov}\left({\tilde{z}}_{1},{\tilde{z}}_{1}\right)=\sum_{l=1}^{L}E\left({\tilde{G}}_{l}{\tilde{G}}_{l}^{H}\right)+\gamma_{U}I_{M}, \end{array}$$

and $E\left({\tilde{G}}_{l}{\tilde{G}}_{l}^{H}\right)$ can be obtained from Equation (8).

Equation (9) can be viewed as the equivalent system model of Equation (1) with channel estimation error. With the well-known properties of MMSE estimate, one obtains

$$\mathrm{cov}\left({\hat{G}}_{l}x_{l},{\tilde{z}}_{1}\right)=0,$$

which means ${\tilde{z}}_{1}$ is uncorrelated additive noise. We define the “effective SNR” as $1/\gamma_{\mathrm{eff}}$, where

$$\gamma_{\mathrm{eff}}=\frac{1}{M}\mathrm{Tr}\left(\Sigma \right).$$

From Equation (8), we can see that even if both $\gamma_{P}$ and $\gamma_{U}$ become zero, $\gamma_{\mathrm{eff}}$ cannot be zero.

## 3. Sum-Rate Analysis for MU-MIMO with Multi-Cell Pilot Contamination

In this section, we first present the lower bound of the sum-rate for the equivalent system model in Equation (9). Then, the closed-form expression of the moment generating function (MGF) of the lower bound is derived. With the MGF of the sum-rate, the first two moments of the sum-rate are obtained. Furthermore, with Gaussian approximation, we give the closed-form expressions for the outage rate and the outage probability of the sum-rate. Finally, considering BS with very large number of antennas, the asymptotic mean and variance of the sum-rate are derived by using random matrix theory.

### 3.1. Lower Bound of Sum-Rate

Given estimated channel knowledge ${\hat{G}}_{1},\ldots ,{\hat{G}}_{L}$, the sum rate of cell 1 in nats per second per channel use (nats/s/channel) is denoted as $I\left(x_{1};y_{1}|{\hat{G}}_{1},\ldots ,{\hat{G}}_{L}\right)$ [17], where I(·;·) denotes the mutual information.

**Theorem** **1.** *The lower bound of $I\left(x_{1};y_{1}|{\hat{G}}_{1},\ldots ,{\hat{G}}_{L}\right)$, denoted as C, can be given by*

(11) $$\begin{array}{c} C=\ln\det\left[\gamma_{1}{\hat{H}}^{H}\Xi \hat{H}{\left(\gamma_{2}{\hat{H}}^{H}\Xi \hat{H}+I_{K}\right)}^{-1}+I_{K}\right], \end{array}$$

*where*

$$\gamma_{1}≜\lambda_{1}^{2},\;\gamma_{2}≜\sum_{l=2}^{L}\lambda_{l}^{2},$$

*and*

$$\Xi ≜R^{2}{\left(Q\Sigma \right)}^{-1}.$$

**Proof.**  See Appendix A. □

**Remark** **1.** *In Theorem 1, the lower bound of the sum-rate for MU-MIMO systems under correlated Rayleigh fading is given. Based on Equation (11), we can further analyze the statistical characteristics of C. Note that imperfect CSI and multi-cell pilot contamination are taken into account in Theorem 1. Thus, the derived lower bound of the mutual information is different from that in [14], which considers perfect channel estimation and no pilot contamination.*

### 3.2. Derivation of MGF for the Lower Bound of Sum-Rate

Without loss of generality, we assume that R is a full rank matrix and has *M* distinct eigenvalues. Then, we see that Ξ is also a full rank matrix with *M* distinct eigenvalues.

**Theorem** **2.** *The entries of $\hat{H}$ are i.i.d. ZMCSCG with unit variance. Let **Ξ** be an M×M positive definite matrix, the M distinct ordered eigenvalues of $\Xi^{-1}$ are $0<\xi_{M}<\xi_{M-1}<\ldots <\xi_{1}<\infty$. Let $W={\hat{H}}^{H}\Xi \hat{H}$ and*

$$C=\ln\det\left[\gamma_{1}W{\left(\gamma_{2}W+I_{K}\right)}^{-1}+I_{K}\right].$$

*The MGF of C is*

(12) $$\begin{array}{cc} M_{C}\left(\upsilon \right) & ≜E\left(e^{\upsilon C}\right)=T\det\left[\Theta \left(\upsilon \right)\right], \end{array}$$

*where*

$$T=\frac{{\left(-1\right)}^{K\left(M-K\right)}}{\Gamma_{\left(K\right)}\left(K\right)}\frac{\prod_{m=1}^{M}\xi_{m}^{K}}{\prod_{i<j}\left(\xi_{i}-\xi_{j}\right)},$$

*$\Gamma_{\left(K\right)}\left(K\right)\overset{\Delta }{\;=}\prod_{k=1}^{K}\left(K-k\right)!$, $\Theta \left(\upsilon \right)$ is M×M matrix whose (i,j)th entry is given by*

$${\left[\Theta \left(\upsilon \right)\right]}_{i,j}=\left\{\begin{array}{cc} J\left(\xi_{i},j,\gamma_{1},\gamma_{2},\upsilon \right) & j=1,\ldots ,K\\ \xi_{i}^{M-j} & j=K+1,\ldots ,M \end{array}\right..$$

*After tedious algebra, $J\left(\xi_{i},j,\gamma_{1},\gamma_{2},\upsilon \right)$ can be evaluated by*

(13) $$\begin{array}{c} J\left(\xi_{i},j,\gamma_{1},\gamma_{2},\upsilon \right)\\ =\int_{0}^{\infty }x^{j-1}e^{-\xi_{i}x}{\left(1+\frac{\gamma_{1}x}{1+\gamma_{2}x}\right)}^{\upsilon }dx\\ ={\left(\gamma_{2}\right)}^{-\upsilon }{\left(\gamma_{1}+\gamma_{2}\right)}^{\upsilon -j}\exp\left(\frac{1}{\gamma_{1}+\gamma_{2}}\xi_{i}\right)\\ \;\times \sum_{n=0}^{j-1}\left(\begin{array}{c} j-1\\ n \end{array}\right){\left(-1\right)}^{j-1-n}{\left(\frac{\gamma_{1}}{\gamma_{2}}\right)}^{n+1}\Gamma \left(n+\upsilon +1\right)\\ \;\times U\left(n+\upsilon +1,n+2,\frac{\gamma_{1}\xi_{i}}{\left(\gamma_{1}+\gamma_{2}\right)\gamma_{2}}\right) \end{array}$$

*where $\Gamma \left(\cdot \right)$ is the standard Gamma function and $U\left(a,b,z\right)$ is the confluent hypergeometric function of the second kind [18] (eq. (9.211.4)).*

**Proof.**  See Appendix B. □

**Remark** **2.** *In Theorem 2, the closed-form expression of MGF for the sum-rate is given, with which we can derive the approximated first two moments of the sum-rate, i.e., ergodic sum-rate and variance of the sum-rate, in closed-form.*

### 3.3. Ergodic Sum-Rate

With the MGF of *C*, the *u*th moment of *C*, $E\left(C^{u}\right)$, can be obtained by

(14) $$\begin{array}{c} E\left(C^{u}\right)={\left.\frac{\partial^{u}M_{C}\left(\upsilon \right)}{\partial \upsilon^{u}}\right|}_{\upsilon =0}=T{\left.\frac{\partial^{u}\det\left[\Theta \left(\upsilon \right)\right]}{\partial \upsilon^{u}}\right|}_{\upsilon =0}. \end{array}$$

To compute Equation (14), we can make use of the following formula [19] (eq. (6.1.19)),

(15) $$\begin{array}{c} \frac{\partial \det\left[\Theta \left(\upsilon \right)\right]}{\partial \upsilon }=\sum_{m=1}^{M}\det\left[\Theta_{m}\left(\upsilon \right)\right], \end{array}$$

where $\Theta_{m}\left(\upsilon \right)$ is identical to $\Theta \left(\upsilon \right)$ except that the entries in the *m*th column are replaced by their derivatives.

Then, for u=1, we have

(16) $$\begin{array}{c} E\left(C\right)=T\sum_{m=1}^{M}\det\left(\Theta_{m}\right), \end{array}$$

where $\Theta_{m}$ are M×M matrices with entries

(17) $$\begin{array}{c} {\left[\Theta_{m}\right]}_{i,j}=\left\{\begin{array}{c} P_{1}\left(\xi_{i},\gamma_{1}+\gamma_{2},j\right)-P_{1}\left(\xi_{i},\gamma_{2},j\right),\;j=m\\ P_{0}\left(\xi_{i},j\right),\;\;\;\;\;\;\;j=1,\ldots ,K,j\neq m\\ \xi_{i}^{M-j},\;\;\;\;\;\;\;\;j=K+1,\ldots ,M \end{array}\right. \end{array}$$

for m=1,…,K and

(18) $$\begin{array}{c} {\left[\Theta_{m}\right]}_{i,j}=\left\{\begin{array}{c} 0,\;\;\;\;\;\;\;\;\;\;\;j=m\\ P_{0}\left(\xi_{i},j\right),\;\;\;\;\;\;j=1,\ldots ,K\\ \xi_{i}^{M-j},\;j=K+1,\ldots ,M,j\neq m \end{array}\right. \end{array}$$

for m=K+1,…,M. $P_{0}\left(a,t\right)$ is defined as

$$P_{0}\left(a,t\right)=\int_{0}^{\infty }e^{-ax}x^{t-1}dx,$$

and $P_{1}\left(a,b,t\right)$ defined as

$$P_{1}\left(a,b,t\right)=\int_{0}^{\infty }e^{-ax}x^{t-1}\ln\left(1+bx\right)dx$$

can be evaluated by using [20] (eq. (78)).

### 3.4. Approximated Variance of Sum-Rate

Similarly, with Equation (15), we can compute the second derivative of $\det\left[\Theta \left(\upsilon \right)\right]$ as

$$\frac{\partial^{2}\det\left[\Theta \left(\upsilon \right)\right]}{\partial \upsilon^{2}}=\sum_{m^{\prime }=1}^{M}\sum_{m=1}^{M}\det\left[\Theta_{m,m^{\prime }}\left(\upsilon \right)\right],$$

where $\Theta_{m,m^{\prime }}\left(\upsilon \right)$ is identical to $\Theta_{m}\left(\upsilon \right)$ except that the entries in the $m^{\prime }$th column are replaced by their derivatives.

Then, the second moment of *C* can be expressed as

(19) $$\begin{array}{c} E\left(C^{2}\right)=T\sum_{m^{\prime }=1}^{M}\sum_{m=1}^{M}\det\left(\Theta_{m,m^{\prime }}\right), \end{array}$$

where $\Theta_{m,m^{\prime }}$ are M×M matrices with entries

(20) $$\begin{array}{c} {\left[\Theta_{m,m^{\prime }}\right]}_{i,j}=\left\{\begin{array}{ccc} P_{2}\left(\xi_{i},\gamma_{1}+\gamma_{2},j\right)+P_{2}\left(\xi_{i},\gamma_{2},j\right)-2Q\left(\xi_{i},\gamma_{1}+\gamma_{2},\gamma_{2},j\right), & & j=m\;=m^{\prime }\\ P_{1}\left(\xi_{i},\gamma_{1}+\gamma_{2},j\right)-P_{1}\left(\xi_{i},\gamma_{2},j\right), & & j=m\;\mathrm{or}\;j=m^{\prime };m\neq m^{\prime }\\ P_{0}\left(\xi_{i},j\right), & & j=1,\ldots ,K;j\neq m;j\neq m^{\prime }\\ \xi_{i}^{M-j}, & & j=K+1,\ldots ,M \end{array}\right. \end{array}$$

for $m,m^{\prime }=1,\ldots ,K$ and

(21) $$\begin{array}{c} {\left[\Theta_{m,m^{\prime }}\right]}_{i,j}=\left\{\begin{array}{c} P_{1}\left(\xi_{i},\gamma_{1}+\gamma_{2},j\right)-P_{1}\left(\xi_{i},\gamma_{2},j\right),\;j=m\\ 0,\;\;\;\;\;\;\;\;\;\;\;j=m^{\prime }\\ P_{0}\left(\xi_{i},j\right),\;\;\;\;\;\;j=1,\ldots ,K;j\neq m\\ \xi_{i}^{M-j},\;\;\;\;\;\;j=K+1,\ldots ,M;j\neq m^{\prime } \end{array}\right. \end{array}$$

for $m=1,\ldots ,K,m^{\prime }=K+1,\ldots ,M$ and

(22) $$\begin{array}{c} {\left[\Theta_{m,m^{\prime }}\right]}_{i,j}=\left\{\begin{array}{c} P_{0}\left(\xi_{i},j\right),\;\;\;\;j=1,\ldots ,K\\ 0,\;\;\;\;\;\;\;j=m=m^{\prime }\\ 0,\;\;\;\;\;j=m\;\mathrm{or}\;j=m^{\prime };m\neq m^{\prime }\\ \xi_{i}^{M-j},\;j=K+1,\ldots ,M;j\neq m;j\neq m^{\prime } \end{array}\right. \end{array}$$

for $m=K+1,\ldots ,M,m^{\prime }=K+1,\ldots ,M$. $P_{2}\left(a,b,t\right)$ defined as

$$P_{2}\left(a,b,t\right)=\int_{0}^{\infty }e^{-ax}x^{t-1}\ln^{2}\left(1+bx\right)dx$$

can be evaluated by using [21] (eq. (41)). $Q\left(a,b,c,t\right)$ defined as

$$Q\left(a,b,c,t\right)=\int_{0}^{\infty }e^{-ax}x^{t-1}\ln\left(1+bx\right)\ln\left(1+cx\right)dx$$

can be evaluated with Meijer G-function [22], which can be expressed as

$$Q\left(a,b,c,t\right)=a^{-t}G_{1,0:2,2:2,2}^{0,1:1,2:1,2}\left(\left.{}_{-}^{1-t}\right|\left.{}_{1,0}^{1,1}\right|\left.{}_{1,0}^{1,1}\right|\frac{b}{a},\frac{c}{a}\right).$$

Finally, using Equations (16) and (19), the variance of *C* can be computed as

(23) $$V\left(C\right)=E\left(C^{2}\right)-{\left[E\left(C\right)\right]}^{2}.$$

**Remark** **3.** *In general, the exact distribution of C cannot be obtained even for i.i.d. Rayleigh fading channel. However, based on Monte-Carlo simulation, we observe that the probability density function (PDF) of C resembles that of a Gaussian random variable. Therefore, we approximate the distribution of sum-rate by a Gaussian distribution with equal mean and variance, which are presented in Equations (16) and (23). Note that Gaussian approximation has also been widely used to approximate the distribution of the mutual information for MIMO channels [14,23,24].*

### 3.5. Outage Performance of the Sum-Rate

In practice, outage capacity is another important parameter for wireless communication systems. Thus, we concern to the cumulative distribution function (CDF) of *C*.

The Gaussian-approximated CDF of *C* can now be derived as

(24) $$\mathrm{Pr}\left(C\leq y\right)=1-\frac{1}{2}\mathrm{erfc}\left[\frac{y-E\left(C\right)}{\sqrt{2V\left(C\right)}}\right].$$

Equivalently, the approximated maximum rate at the outage probability *q* (i.e., $\mathrm{Pr}(C<C^{q})=q$) can be given by

(25) $$\begin{array}{cc} C^{q} & =E\left(C\right)+\sqrt{2V\left(C\right)}{\mathrm{erfc}}^{-1}\left(2\left(1-q\right)\right). \end{array}$$

### 3.6. Derivation of the Variance of Sum-Rate for Very Large Number of Antennas

The following theorem shows that *C* asymptotically has a normal distribution. This is not surprising, because, according to Theorem 1 in [14], the distribution of the right-hand-side term of Equation (11) is asymptotically normal as M→∞. However, what is perhaps surprising is the speed with which the variance of *C* decreases with increasing *M*.

**Theorem** **3.** *As M→∞, the sum-rate C in Equation (11) obeys*

(26) $$\begin{array}{cc} & \frac{M^{2}}{\sqrt{K\mathrm{Tr}\left(\Xi^{2}\right)}}\left[C-K\ln\left(1+\frac{M\gamma_{1}\omega }{1+M\gamma_{2}\omega }\right)\right]\\ & \;\;\;\overset{d}{\underset{\;\;\;}{\to }}N\left(0,\frac{{\gamma_{1}}^{2}}{\omega^{4}{\left(\gamma_{1}+\gamma_{2}\right)}^{2}{\gamma_{2}}^{2}}\right), \end{array}$$

*where ω is defined as $\omega ≜\frac{\mathrm{Tr}\left(\Xi \right)}{M}$. A numerically accurate approximation of the variance of C for large M is given by*

(27) $$\begin{array}{c} V\left(C\right)=\frac{{\gamma_{1}}^{2}KTr\left(\Xi^{2}\right)}{{\left[1+\left(\gamma_{1}+\gamma_{2}\right)\omega M\right]}^{2}{\left(1+\gamma_{2}\omega M\right)}^{2}}. \end{array}$$

**Proof.**  See Appendix C. □

**Remark** **4.** *Based on Hypothesis 1, as M→∞, $Tr\left(\Xi^{2}\right)$ increases as M, which is the same as $Tr\left(\Xi \right)$. Thus, Theorem 3 shows that, as the number of antennas increases, the variance of C decreases as $1/M^{3}$. This is different from the point-to-point MIMO channel with perfect CSI. As shown in [14], for the M×K i.i.d. Rayleigh fading MIMO channel, the variance of mutual information decreases as 1/M for large M and fixed K.*

## 4. Numerical Results

The theoretical analysis presented in the last section was validated through a set of Monte-Carlo simulations. First, we generated the related system parameters including large-scale information, correlation matrix and Rayleigh fading channel. Then, based on Equation (11), which describes the sum-rate of the equivalent system model, we calculated the simulation value of system capacity. The simulation results were obtained by averaging over 10,000 correlated Rayleigh block-fading channels.

Similar to [25], a seven-cell system layout was adopted. The inner cell radius *D* was normalized to one, the distance between two adjacent cells was normalized to $\sqrt{3}$, and the polar coordinates of the *L* base stations were at

$$\left(0,0\right),\;\left(\sqrt{3},\frac{2\pi l}{L-1}\right),\;l=1,2,\ldots ,L-1\;.$$

We assumed a distance-based path loss model with path loss exponent $\alpha_{l}=3.7$ for l=1,2,…,L and c=1. In Cell 1, we distributed *K* users uniformly on a circle of radius 2/3 around BS. In other cells, *K* users were distributed around BS. We did not consider shadowing. We further assumed $\gamma_{P}=\gamma_{U}$. For simplicity, the entries of the correlation matrix R were modeled via the common exponential correlation model ${\left[R\right]}_{m,n}=\rho^{\left|m-n\right|}$ with $\rho \in \left[0,1\right)$ being the transmit correlation coefficient [26].

**Figure 2:**
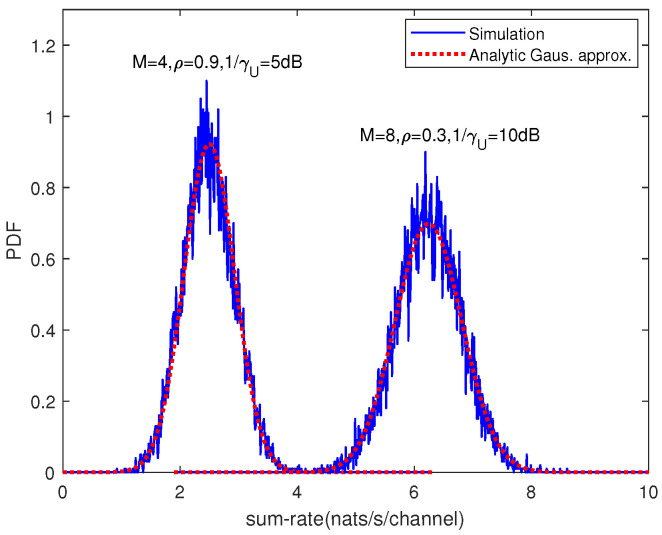
Empirical distribution and Gaussian approximation to the probability density function (PDF) of the sum-rate for various channels with K=4.

First, we considered not-so-large number of antennas. Figure 2 gives the analytical Gaussian approximation to the sum-rate PDF, as well as the empirically generated PDF. The mean and variance of sum-rate for correlated channel were given by Equations (16) and (23), respectively. We can see that the approximation of the mean and variance was satisfied at high SNR.

**Figure 3:**
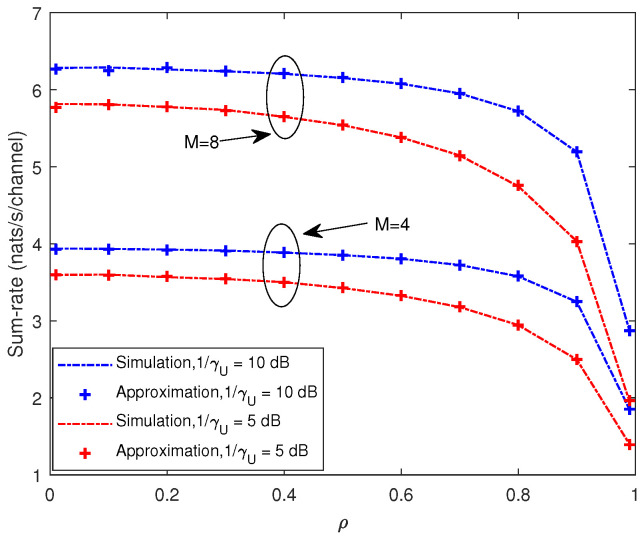
Analytic approximation and simulation results for mean of the sum-rate with K=4.

In Figure 3, the sum-rate is plotted against the correlation coefficient ρ for different values of *M* and $1/\gamma_{U}$ with K=4. The sum-rate decreased as ρ increased. Especially when ρ was large, the sum-rate dropped dramatically. In addition, the sum-rate increased as the number of antennas or the SNR grew.

**Figure 4:**
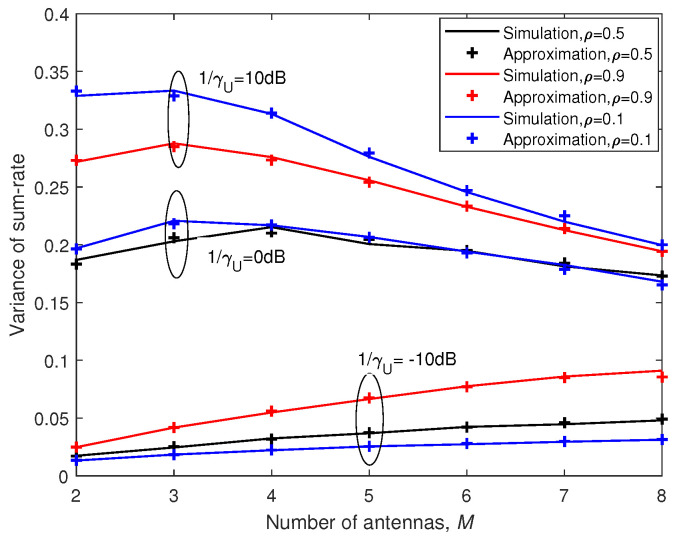
Analytic approximation and simulation results for variance of the sum-rate with K=2.

In Figure 4, the variance of the sum-rate is plotted against the number of antennas *M*. It can be seen that there was no monotonous relation between variance of the sum-rate and *M*. The variance of the sum-rate increased as *M* grew when SNR was small such as $1/\gamma_{U}=-10$ dB, while it first increased and then decreased as *M* increased when SNR was large. In addition, it is shown that variance of the sum-rate became larger as SNR increased generally. Furthermore, we can conclude that there was no monotonous relation between variance of the sum-rate and correlation coefficient ρ.

It can be seen in Figure 3 and Figure 4 that our closed-form approximations were almost indistinguishable from the simulation results over the entire range of ρ for different *M*, *K* and SNR.

**Figure 5:**
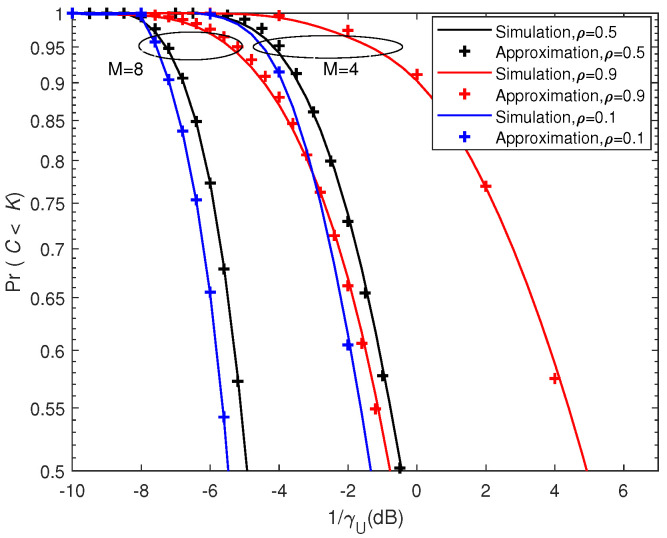
Comparison between the analytic outage probability and simulated outage probability against signal-to-noise ratio (SNR) with K=2.

**Figure 6:**
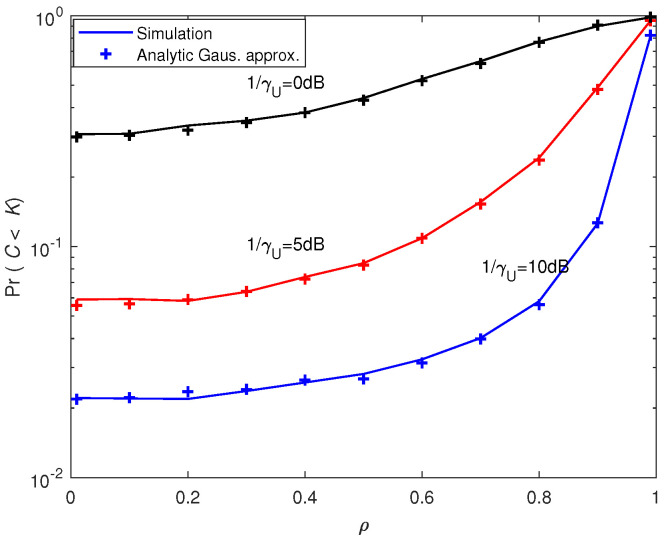
Comparison between the analytic outage probability and simulated outage probability against ρ with M=4 and K=2.

**Figure 7:**
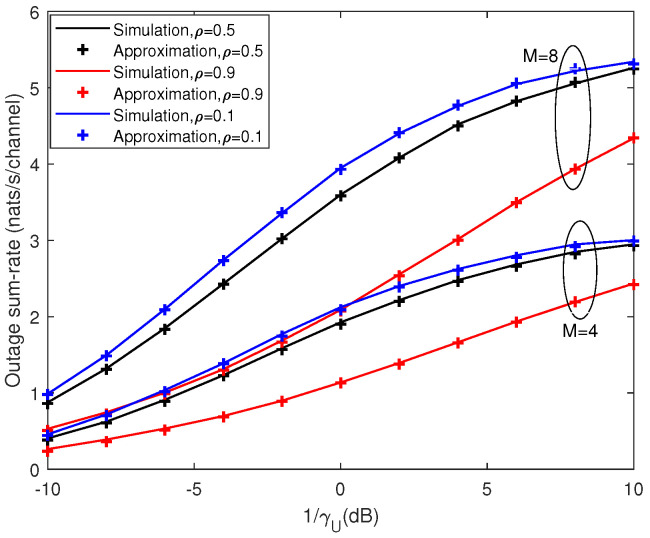
Analytic Gaussian approximation and simulation results for outage sum-rate with K=4. Outage probability is q=0.05.

Figure 5 and Figure 6 depict the outage probability against the SNR and correlation coefficient, respectively. We can see that outage probability decreased as SNR increased, *M* became larger or ρ decreased. When the number of antennas *M* was doubled, the SNR performance gain was more than 4 dB (e.g., for $\mathrm{Pr}\left(C<K\right)=0.75$ in Figure 5). Figure 7 presents the outage sum-rate against the SNR for different ρ and *M* with outage probability q=0.05 and K=4. It can be seen from the figure that the outage sum-rate grew larger when the SNR increased, the number of antennas became bigger or the correlation coefficient decreased. When *M* was doubled, the improvement of the outage sum-rate was more than 75% (e.g., for SNR=10 dB and ρ=0.1 in Figure 7).

**Figure 8:**
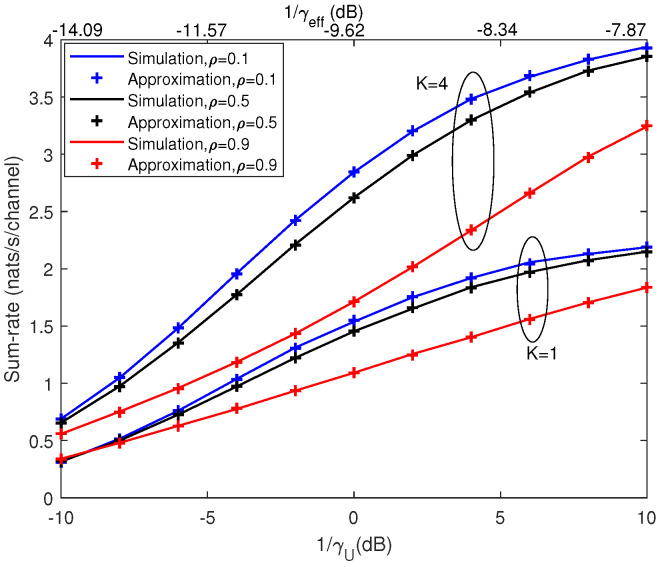
Comparison of mean of the sum-rate under multi-user case and single-user case with M=4.

**Figure 9:**
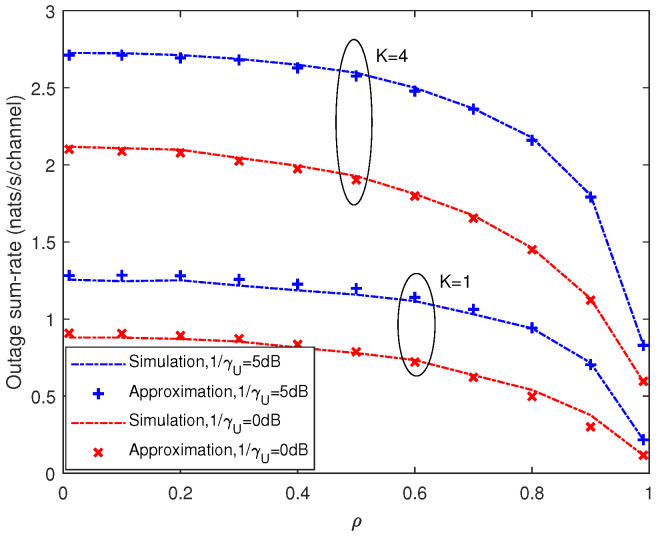
Comparison of outage sum-rate under multi-user case and single-user case with M=4. Outage probability is q=0.05.

Figure 8 and Figure 9 depict the mean of the sum-rate and the outage probability of both multi-user system and single-user system, respectively. Under the same system parameters, the single-user system achieved smaller sum-rate and outage sum-rate compared with that of the multi-user case. For example, in Figure 8, when ρ=0.1 and $1/\gamma_{U}=10$ dB, the achievable sum-rate of single-user system was almost half that of the system with K=4. In Figure 9, when ρ=0.2 and $1/\gamma_{U}=5$ dB, the outage sum-rate of single-user system was even smaller than half that of the system with K=4. Furthermore, the corresponding effective SNR with K=4 and ρ=0.1 is also shown on the top x-axis in Figure 8. It can be seen that, due to pilot contamination, the effective SNR was much smaller than SNR, and, for high SNR, the increasing of the effective SNR became very slow with increasing SNR.

**Figure 10:**
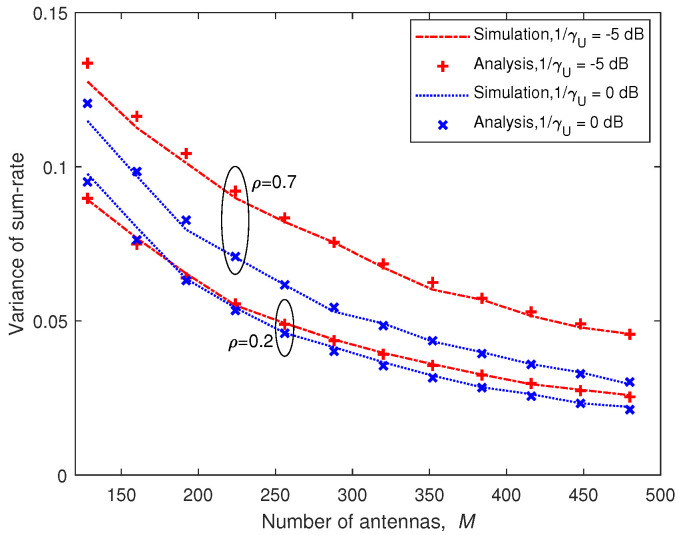
Analytic approximation and simulation results for variance of the sum-rate with K=16 for large *M*.

For a large number of antennas, we considered K=16. Figure 10 illustrates the variance of the sum-rate for large *M*. We can see that the approximation for large *M* was very accurate even for large correlation coefficient. We also see that the degradation due to the high correlation was very large for massive MIMO. Furthermore, as the number of antennas *M* grew, the variance shrank very quickly. This effect is called “channel hardening” [14], which is beneficial for voice and other traffic that is sensitive to channel fluctuations and delay.

## 5. Conclusions

In this paper, we have presented the theoretical analysis of the sum-rate for MU-MIMO systems with multi-cell pilot contamination under correlated Rayleigh fading channels. With joint multi-cell channel estimation and the equivalent system model, we derived the lower bound of the sum-rate. Then, the closed-form expression of the MGF of the lower bound is obtained. With Gaussian approximation, we derived the first two moments of the lower bound and the outage performance of the sum-rate. We also investigated the asymptotic performance of the sum-rate when BS is equipped with very large number of antennas. Simulation results have verified the accuracy of the analytical approximation and the performance degradation due to the approximation is negligible.

## Appendix A. Proofs of Theorem 1

According to Theorem 1 in [27], the lower bound of $I\left(x_{1};y_{1}|{\hat{G}}_{1},\ldots ,{\hat{G}}_{L}\right)$, which is denoted as *C*, can be given by

(A1) $$\begin{array}{c} C=\ln\det\left(\sum_{l=1}^{L}{\hat{G}}_{l}{\hat{G}}_{l}^{H}+\Sigma \right)-\ln\det\left(\sum_{l=2}^{L}{\hat{G}}_{l}{\hat{G}}_{l}^{H}+\Sigma \right). \end{array}$$

Alternatively, Equation (A1) can be written as

(A2) $$\begin{array}{cc} C= & \ln\det\left(\Sigma^{-1}\sum_{l=1}^{L}{\hat{G}}_{l}{\hat{G}}_{l}^{H}+I_{M}\right)\\ & -\ln\det\left(\Sigma^{-1}\sum_{l=2}^{L}{\hat{G}}_{l}{\hat{G}}_{l}^{H}+I_{M}\right). \end{array}$$

Substituting ${\hat{G}}_{l}$ in Equation (A2) with Equation (7), and using the following matrix identity

(A3) $$\begin{array}{c} \det\left(I+\mathrm{AB}\right)=\det\left(I+\mathrm{BA}\right), \end{array}$$

we obtain

(A4) $$\begin{array}{cc} C & =\ln\det\left[\left(\sum_{l=1}^{L}\lambda_{l}^{2}\right){\hat{H}}^{H}Q^{-\frac{1}{2}}R\Sigma^{-1}RQ^{-\frac{1}{2}}\hat{H}+I_{K}\right]\\ & -\ln\det\left[\left(\sum_{l=2}^{L}\lambda_{l}^{2}\right){\hat{H}}^{H}Q^{-\frac{1}{2}}R\Sigma^{-1}RQ^{-\frac{1}{2}}\hat{H}+I_{K}\right]. \end{array}$$

Since R, Q, and Σ have the same eigenvectors, we have

$$Q^{-\frac{1}{2}}R\Sigma^{-1}RQ^{-\frac{1}{2}}=R^{2}{\left(Q\Sigma \right)}^{-1}.$$

Using the following matrix identity for an invertible matrix A,

(A5) $$\begin{array}{c} \det\left(I+BA^{-1}\right)=\det\left(A+B\right)-\det\left(A\right), \end{array}$$

we can obtain the final result.

## Appendix B. Proofs of Theorem 2

Let $\phi_{1}\geq \phi_{2}\geq \ldots \geq \phi_{K}\geq 0$ be *K* distinct ordered eigenvalues of W. Then, *C* can be written as

(A6) $$\begin{array}{c} C=\ln\left[\prod_{k=1}^{K}\Phi \left(\phi_{k}\right)\right], \end{array}$$

where

$$\Phi \left(x\right)=\frac{1+\left(\gamma_{1}+\gamma_{2}\right)x}{1+\gamma_{2}x}.$$

The joint PDF of the (real) ordered eigenvalues of W is given by [28]

(A7) $$\begin{array}{c} f_{\phi }\left(x_{1},\ldots ,x_{K}\right)=T\det\left[V\left(x\right)\right]\det\left[F\left(x,\xi \right)\right], \end{array}$$

where

$$T=\frac{{\left(-1\right)}^{K\left(M-K\right)}}{\Gamma_{\left(K\right)}\left(K\right)}\frac{\prod_{m=1}^{M}\xi_{m}^{K}}{\prod_{i<j}\left(\xi_{i}-\xi_{j}\right)},$$

$V\left(x\right)$ is Vandermonde matrix with entries ${\left[V\left(x\right)\right]}_{i,j}=x_{j}^{i-1}$ and

$${\left[F\left(x,\xi \right)\right]}_{i,j}=\left\{\begin{array}{cc} e^{-\xi_{i}x_{j}} & j=1,\ldots ,K\\ \xi_{i}^{M-j} & j=K+1,\ldots ,M \end{array}\right..$$

Then, the MGF of *C*, $M_{C}\left(\upsilon \right)$, can be computed as

(A8) $$\begin{array}{c} M_{C}\left(\upsilon \right)=E\left(e^{\upsilon C}\right)=E{\left[\prod_{k=1}^{K}\Phi \left(\phi_{k}\right)\right]}^{\upsilon }. \end{array}$$

Utilizing the joint PDF of the eigenvalues of W given in Equation (A7), we obtain

(A9) $$\begin{array}{cc} M_{C}\left(\upsilon \right) & =T\int \ldots \int_{D_{\mathrm{ord}}}\det\left[V\left(x\right)\right]\det\left[F\left(x,\xi \right)\right]\\ & \;\times {\left[\prod_{k=1}^{K}\Phi \left(x_{k}\right)\right]}^{\upsilon }dx_{1}\ldots dx_{K}, \end{array}$$

where the multiple integral is over the domain

$$D_{\mathrm{ord}}=\left\{\infty >x_{1}\geq x_{2}\geq \ldots \geq x_{K}\geq 0\right\}.$$

Since the integrand is symmetric in $x_{1},x_{2},\ldots ,x_{K}$, according to [29] (Corollary 2), we obtain the result shown in Equation (12).

## Appendix C. Proofs of Theorem 3

According to strong law of large numbers, we have $\frac{1}{M}W\overset{a.s.}{\to }\frac{\mathrm{Tr}\left(\Xi \right)}{M}I_{K}$ as M→∞. Define $\omega ≜\frac{\mathrm{Tr}\left(\Xi \right)}{M}$ and $\frac{\phi_{k}}{M}≜\omega +{\tilde{\phi }}_{k}$. With Equation (A6), we can express *C* in terms of ω and ${\tilde{\phi }}_{k}$ as

(A10) $$\begin{array}{cc} C & =K\ln\left(1+\frac{M\gamma_{1}\omega }{1+M\gamma_{2}\omega }\right)\\ & \;+\sum_{k=1}^{K}\ln\left[1+\frac{M\left(\gamma_{1}+\gamma_{2}\right){\tilde{\phi }}_{k}}{1+M\left(\gamma_{1}+\gamma_{2}\right)\omega }\right]\\ & \;-\sum_{k=1}^{K}\ln\left(1+\frac{M\gamma_{2}{\tilde{\phi }}_{k}}{1+M\gamma_{2}\omega }\right). \end{array}$$

According to strong law of large numbers, we have ${\tilde{\phi }}_{k}\overset{a.s.}{\to }0$ as M→∞. As M→∞, consider the following equation

(A11) $$\begin{array}{cc} & \frac{M^{2}}{\sqrt{K\mathrm{Tr}\left(\Xi^{2}\right)}}\left[C-K\ln\left(1+\frac{M\gamma_{1}\omega }{1+M\gamma_{2}\omega }\right)\right]\\ & =J_{1}\frac{M}{\sqrt{K\mathrm{Tr}\left(\Xi^{2}\right)}}\sum_{k=1}^{K}{\tilde{\phi }}_{k}+J_{2}O\left(\frac{M}{\sqrt{K\mathrm{Tr}\left(\Xi^{2}\right)}}\sum_{k=1}^{K}{\tilde{\phi }}_{k}^{2}\right), \end{array}$$

where $J_{1}$ and $J_{2}$ are given by

$$J_{1}=M\left[\frac{\gamma_{1}+\gamma_{2}}{1/M+\left(\gamma_{1}+\gamma_{2}\right)\omega }-\frac{\gamma_{2}}{1/M+\gamma_{2}\omega }\right],$$

$$J_{2}=\frac{M}{2}\left\{\frac{\gamma_{2}^{2}}{{\left(1/M+\gamma_{2}\omega \right)}^{2}}-\frac{{\left(\gamma_{1}+\gamma_{2}\right)}^{2}}{{\left[1/M+\left(\gamma_{1}+\gamma_{2}\right)\omega \right]}^{2}}\right\},$$

respectively, and both $J_{1}$ and $J_{2}$ are O. Here, we say that $x_{N}=O\left(y_{N}\right)$ if $\left|x_{N}\right|\leq a\left|y_{N}\right|$ for some a>0 and all *N* are sufficiently large.

As shown in [14], $\frac{M}{\sqrt{K\mathrm{Tr}\left(\Xi^{2}\right)}}\sum_{k=1}^{K}{\tilde{\phi }}_{k}$ is the dominant random variable in Equation (A11). According to [14] (Lemma 2), we have

$$\frac{M}{\sqrt{K\mathrm{Tr}\left(\Xi^{2}\right)}}\sum_{k=1}^{K}{\tilde{\phi }}_{k}\overset{a.s.}{\to }N\left(0,1\right).$$

Because, as M→∞,

$$J_{1}\overset{a.s.}{\to }\frac{\gamma_{1}}{\omega^{2}\left(\gamma_{1}+\gamma_{2}\right)\gamma_{2}},$$

we conclude that

$$\begin{array}{c} \frac{M^{2}}{\sqrt{K\mathrm{Tr}\left(\Xi^{2}\right)}}\left[C-K\ln\left(1+\frac{M\gamma_{1}\omega }{1+M\gamma_{2}\omega }\right)\right]\\ \;\;\;\overset{d}{\underset{\;\;\;}{\to }}N\left(0,\frac{{\gamma_{1}}^{2}}{\omega^{4}{\left(\gamma_{1}+\gamma_{2}\right)}^{2}{\gamma_{2}}^{2}}\right). \end{array}$$

Finally, based on Equation (A11), the variance of *C* can be approximated as

(A12) $$\begin{array}{cc} V\left(C\right) & =\frac{K\mathrm{Tr}\left(\Xi^{2}\right)}{M^{4}}{J_{1}}^{2}\\ & =\frac{{\gamma_{1}}^{2}K\mathrm{Tr}\left(\Xi^{2}\right)}{{\left[1+\left(\gamma_{1}+\gamma_{2}\right)\omega M\right]}^{2}{\left(1+\gamma_{2}\omega M\right)}^{2}}. \end{array}$$
